# Phyletic Profiling with Cliques of Orthologs Is Enhanced by Signatures of Paralogy Relationships

**DOI:** 10.1371/journal.pcbi.1002852

**Published:** 2013-01-03

**Authors:** Nives Škunca, Matko Bošnjak, Anita Kriško, Panče Panov, Sašo Džeroski, Tomislav Šmuc, Fran Supek

**Affiliations:** 1 ETH Zurich, Computer Science, Zurich, Switzerland; 2 Swiss Institute of Bioinformatics, Zurich, Switzerland; 3 Ruđer Bošković Institute, Division of Electronics, Zagreb, Croatia; 4 Mediterranean Institute for Life Sciences, Split, Croatia; 5 Jozef Stefan Institute, Department of Knowledge Technologies, Ljubljana, Slovenia; 6 Jozef Stefan International Postgraduate School, Ljubljana, Slovenia; 7 Centre of Excellence for Integrated Approaches in Chemistry and Biology of Proteins, Ljubljana, Slovenia; University of Chicago, United States of America

## Abstract

New microbial genomes are sequenced at a high pace, allowing insight into the genetics of not only cultured microbes, but a wide range of metagenomic collections such as the human microbiome. To understand the deluge of genomic data we face, computational approaches for gene functional annotation are invaluable. We introduce a novel model for computational annotation that refines two established concepts: annotation based on homology and annotation based on phyletic profiling. The phyletic profiling-based model that includes both inferred orthologs and paralogs—homologs separated by a speciation and a duplication event, respectively—provides more annotations at the same average Precision than the model that includes only inferred orthologs. For experimental validation, we selected 38 poorly annotated *Escherichia coli* genes for which the model assigned one of three GO terms with high confidence: involvement in DNA repair, protein translation, or cell wall synthesis. Results of antibiotic stress survival assays on *E. coli* knockout mutants showed high agreement with our model's estimates of accuracy: out of 38 predictions obtained at the reported Precision of 60%, we confirmed 25 predictions, indicating that our confidence estimates can be used to make informed decisions on experimental validation. Our work will contribute to making experimental validation of computational predictions more approachable, both in cost and time. Our predictions for 998 prokaryotic genomes include ∼400000 specific annotations with the estimated Precision of 90%, ∼19000 of which are highly specific—e.g. “penicillin binding,” “tRNA aminoacylation for protein translation,” or “pathogenesis”—and are freely available at http://gorbi.irb.hr/.

## Author Summary

While both the number and the diversity of sequenced prokaryotic genomes grow rapidly, the number of specific assignments of gene functions in the databases remains low and skewed toward the model prokaryote *Escherichia coli*. To aid in understanding the full set of newly sequenced genes, we created a computational model for assignment of function to prokaryotic genomes. The result is an innovative framework for orthology and paralogy-aware phyletic profiling that provides a large number of computational annotations with high predictive accuracy in train/test evaluations. Our predictions include annotations for 1.3 million genes with the estimated Precision of 90%; these, and many more predictions for 998 prokaryotic genomes are freely available at http://gorbi.irb.hr/. More importantly, we show a proof of principle that our functional annotation model can be used to generate new biological hypotheses: we performed experiments on 38 *E. coli* knockout mutants and showed that our annotation model provides realistic estimates of predictive accuracy. With this, our work will contribute to making experimental validation of computational predictions more approachable, both in cost and time.

## Introduction

Many computational methods for functional annotation of genes are based on a search for sequences with common evolutionary descent—homologs. One possible encoding of homology is the use of phyletic profiles: each row in the phyletic profile represents one gene, and the columns represent the presence or absence of homologs in sequenced genomes [1], [2].

There are two main ways in which phyletic profiles can be used for annotation of gene function. Both of them involve propagating the annotation label. First, one could create phyletic profiles and propagate the annotation label within the profile—from genes with known function to their homologs included in the profile. This is homology-based annotation, and many schemes for doing so are possible [3]. Second, one could propagate labels between the profiles by finding similar profiles: assuming that genes that are inherited together tend to work together, one transfers annotation from a better-studied group of homologs to a profile that is similar but contains genes that are not as well studied. Again, this can be done in many ways. For example, phyletic profiles can be grouped by similarity using a variety of distance measures [e.g.], [ 1,4] possibly involving a machine learning framework [e.g.], [ 4]–[6]. Rows in the phyletic profile can stand for genes or groups of genes [e.g.], [ 1], [7,8]; functional annotation can be assigned using a range of vocabularies, e.g., UniProt controlled vocabulary of keywords [9], Enzyme Commission numbers [10], or arguably the most widespread vocabulary, the Gene Ontology [11]. In addition, one could employ some hybrid between the first two approaches, e.g., when the evidence in favour of within-profile label propagation is used to improve the confidence of between-profile propagation and vice versa.

Refinements of homology-based annotation include making a distinction between two types of homologous relationships: orthologs—sequences derived from the same gene in the last common ancestor, and paralogs—sequences derived from a duplication event [12]. Because orthologous pairs are expected to keep the same function [13]–[15] and paralogous pairs are expected to diverge in function [16], the canonical approach to functional annotation relies on transfer of function between orthologs. However, the latest evidence suggests that, relative to pairs of paralogs, the conservation of function between pairs of orthologs is not as strong as the standard model would imply [17].

Our goal was to create a functional annotation model that learns to associate gene function with specific patterns in phyletic profiles—the presence and absence of different types of homologs in different organisms. To create the phyletic profiles, we combined ortholog cliques—fully interconnected groups of orthologs—with both additional orthologs and additional paralogs. We found that, instead of reducing the predictive accuracy, paralogs provide valuable information: compared to the model that includes only orthologs, the model that includes both orthologs and paralogs gave more predictions at the same average correctness.

In addition, we performed experimental assays in the model organism *Escherichia coli*, showing that the annotation model provides a realistic assessment of confidence for the predicted annotations: a growth phenotype screen on *E. coli* knockout mutants indicated an overall Precision of 66%—out of 38 tested genes, we confirmed predictions for 25—agreeing with the expected Precision of 60%.

We predict Gene Ontology annotations at various levels of specificity for about 1.3 million poorly annotated genes in 998 prokaryotes at a stringent threshold of 90% Precision: about 19000 of those are highly specific functions. In addition to these, we provide many more predictions at less stringent cut-offs in a Web resource GORBI (http://gorbi.irb.hr/).

## Results

We created our functional annotation models in three steps: 1) constructing the phyletic profiles, 2) functionally annotating them where possible, and 3) using a decision tree-based classifier to find groups of profiles that are similar or dissimilar. We detail these steps below.

**Figure 1:**
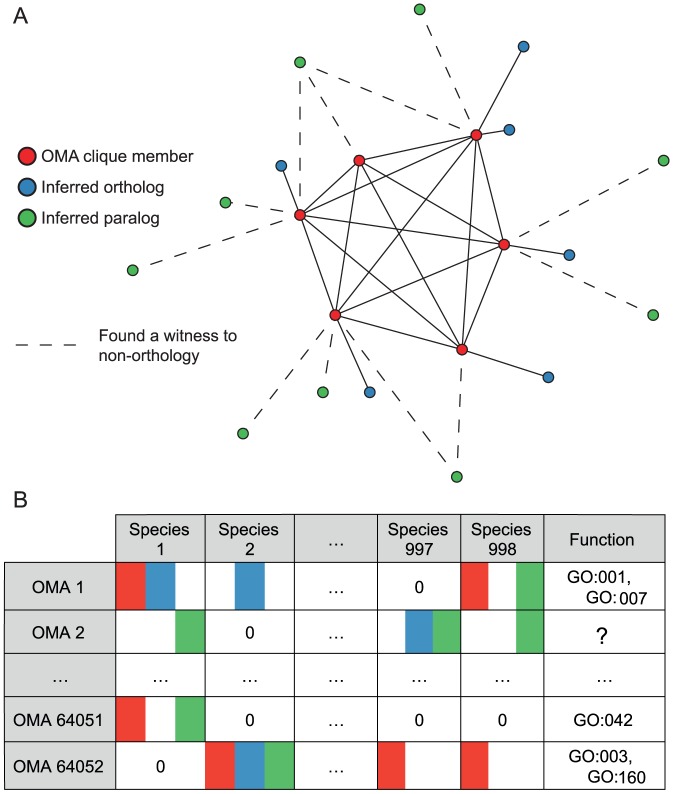
Constructing phyletic profiles with the relations inferred by the OMA algorithm. A) One OMA group and the possible relations used in constructing phyletic profiles: members of an OMA group are all connected by orthologous relations and they form a clique (red); some orthologous proteins were left out in the process of forming cliques because they lack an orthologous connection to at least one group member (blue); a witness to non-orthology infers paralogs (green) [35] B) Constructing phyletic profiles: presence of the corresponding homolog is shown with the colours and their combinations. For example, when constructing the phyletic profile that accounts for OMA clique members (red) and all left out orthologs (blue), the cell in the 1st column and 1st row will have ‘1’: ‘Species 1’ has an ‘OMA 1’ clique member (red) and at least one more protein in an orthologous relationship with at least one protein from ‘OMA 1’ (blue); the cell in the 998th column and 2nd row will have ‘0’: ‘Species 998’ only has protein(s) in a paralogous relationship to ‘OMA 2’ members. In the Function column, the Gene Ontology annotations are assigned when at least half of the OMA clique members have the respective annotation. This figure is featured on the GORBI web site: http://gorbi.irb.hr/en/method/oma-cliques-in-phylogenetic-profiling/.

The first step is constructing the phyletic profiles; in fact, this step is what differentiates between models proposed in this work. To choose among these models for functional annotation, we constructed four kinds of phyletic profiles (Figure 1). First, phyletic profiles of OMA cliques of orthologs: each profile represents the pattern of presence/absence of an OMA clique member among 909 Bacterial and 89 Archaeal genomes. Second, we added the presence patterns for all orthologs inferred by the OMA algorithm that did not participate in the ortholog clique; these orthologous pairs include inferred one-to-one, one-to-many, many-to-one, and many-to-many orthologs. Third, we added presence patterns for all paralogs inferred by the OMA algorithm; these are in fact inferred between-species paralogs—broken pairs in the OMA algorithm. The within-species paralogs are accounted for implicitly: if an OMA clique member is connected to a within-species paralog, the binary phyletic profile does not change. Fourth, we made a separate set of phyletic profiles that only include clique members and paralogs, but not the orthologs outside of the clique.

The second step is annotating the phyletic profiles with a GO term if at least half of clique members had the respective GO term assigned to them. We determined this threshold empirically (see Materials and methods) in order to maximize functional consistency of known annotations within OMA cliques. The additional orthologs and paralogs were not considered in GO term annotation, even when their presence/absence was used in creating the profile. In other words, the difference between the functional annotation models is in the pattern of presence/absence of different types of homologs, and not in the functional annotations assigned to the phyletic profile.

The final step is measuring the (dis)similarity between profiles. We presented both the annotated and the poorly annotated phyletic profiles to a machine learning algorithm based on decision trees. In the decision tree algorithm, the groups of phyletic profiles are recursively divided into subsets based on their presence/absence patterns. In fact, the similarity measure is not defined *a priori*, but is instead inferred from the data: those homologs whose presence/absence best discriminates between GO terms are used to determine which profiles are more similar. In the final step of the decision tree algorithm, the most similar phyletic profiles are placed in leaves: this allows us to propagate the GO term annotation across profiles within these leaves.

Here, we used an algorithm based on decision trees, Clus-HMC-Ens [18]. Clus-HMC-Ens is based on combining multiple decision trees in a Random Forest-like setting [19], and can handle multiple labels—here, GO terms—for each phyletic profile. Furthermore, Clus-HMC-Ens is aware of the hierarchical relationships between the multiple labels and uses this information to improve predictive accuracy [18].

We report three performance measures: Precision, Recall, and Area Under the Precision-Recall Curve (AUPRC). Precision stands for the fraction of predictions that are known to be true, Recall stands for the fraction of known annotations that were successfully predicted, and AUPRC summarizes both Precision and Recall at all possible stringency thresholds of the annotation model. Formal definitions of these measures and the machine-learning algorithm's train/test procedure used to obtain them are detailed in the Materials and methods section.

### Both orthologs and paralogs contribute to predictive accuracy of phyletic profiles

**Figure 2:**
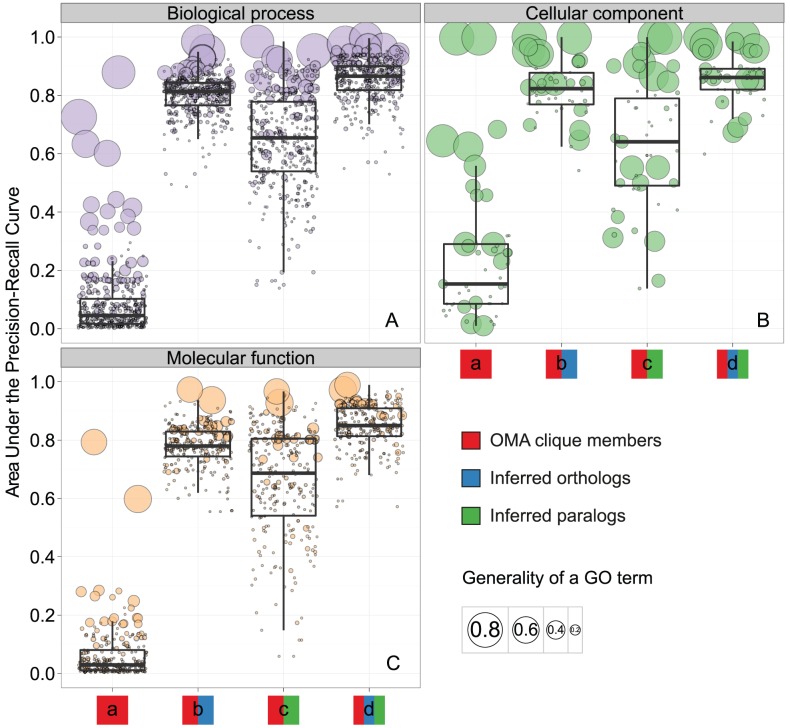
Predictive performance of the four analysed models for the three Gene Ontologies. A) Biological Process, B) Cellular Component, and C) Molecular Function. The x axis represents the models: phyletic profiles are based on (a) OMA cliques of orthologs; (b) OMA cliques of orthologs and OMA inferred orthologs; (c) OMA cliques of orthologs and OMA inferred paralogs; and (d) OMA cliques of orthologs, OMA inferred orthologs, and OMA inferred paralogs. The y-axis represents the Area Under the Precision-Recall Curve (AUPRC). Each disc represents one GO term; its colour represents the ontology, while the area of the disc is proportional to the generality of the GO term: the frequency of the GO term among all annotations available in 07-02-2012 UniProt-GOA release. Each boxplot summarizes AUPRC for the dataset indicated on the x-axis. Lower, mid, and upper horizontal lines denote the first quartile, median and the third quartile, respectively; vertical lines reach 1.5 interquartile range from the respective quartile or the extreme value, whichever is closer.

In one OMA clique, inferred orthologous relations connect each protein to every other protein, so it is not surprising that they group proteins with mostly the same function (see Materials and methods). However, OMA cliques leave out many of the existing orthologous relations. Consequently, phyletic profiles of OMA cliques are incomplete, leading to poor performance in our classification model: many of the true orthologous relations are missing, and the model can successfully annotate using only the most general GO terms (Figure 2, model a; Figure S1 in Text S1: panels A, B, and C). If we compensate for the missing orthologous relations in OMA cliques by adding all inferred one-to-one, one-to-many, many-to-one, and many-to-many orthologs left out when constructing the cliques, the model improves: the mean AUPRC is 0.8 (Figure 2, model b; Figure S1 in Text S1: panels D, E, and F). We also tested whether adding paralogs to phyletic profiles of OMA cliques improves the mean AUPRC: it does, showing that the functional information we obtain from paralogs is far from useless (Figure 2, model c; Figure S1 in Text S1: panels G, H, and I). Still, the mean AUPRC is 0.65—lower than if we enrich phyletic profiles with orthologous relationships. However, it is the combined information from orthologs and paralogs that provides us with the best model for functional annotation (Figure 2, model d; Figure S1 in Text S1: panels J, K, and L): the mean AUPRC increases to 0.85.

In the above experiments, adding only orthologs improved AUPRC more than adding only paralogs (Figure 2, models b and c, respectively). To test whether accounting for the ortholog/paralog distinction would further increase AUPRC, we encoded the phyletic profiles with three levels: presence of an OMA clique member or another ortholog (2), presence of a paralog (1), or absence of any of these (0). We found a small gain in AUPRC resulting from the ortholog/paralog distinction (Figure S2 in Text S1, panel B), but we also found that increasing the number of levels in the dataset from the original two to the above-described three decreases the AUPRC (Figure S2 in Text S1, panel A). Taken together, accounting for the ortholog/paralog distinction did not yield an overall gain in AUPRC in the current machine learning setup (Figure S2 in Text S1, panel C), so we chose the binary model as our principal result.

### Consistent gains in accuracy across GO terms

In this binary model that includes both orthologs and paralogs, most of the general GO terms have high AUPRC. More specific GO terms span a wide range of AUPRC (Figure S3 in Text S1). Nevertheless, both specific and general GO terms benefit from the inclusion of orthologs and paralogs. Specific GO terms such as “lysine biosynthetic process via diaminopimelate,” “organic acid∶sodium symporter activity,” or “bacterial-type flagellum basal body” are used in less than 0.1% of annotations in the 07-02-2012 UniProt-GOA release (their Information Content is higher than 10): the mean AUPRC of this subset of specific GO terms rises from 0.78 in the model that includes orthologs (Figure 2, model b) to 0.83 in the model that includes both orthologs and paralogs (Figure 2, model d). For the general GO terms such as “protein transport,” “kinase activity,” or “plasma membrane,” each used in more than 3% of annotations in the 07-02-2012 UniProt-GOA release (their Information Content is lower than 5), the corresponding change in AUPRC is from 0.80 to 0.88.

Intuitively, phyletic profiling should perform best for the Biological Process (BP) GO terms: proteins with similar profiles are expected to be involved in the same BP but not necessarily to have the same Molecular Function (MF). For example, one kinase and one glucosidase may be involved in the same process of sporulation despite having different MF. As a result, one would expect phyletic profiling to be more appropriate for assigning BP GO terms than MF GO terms.

Here, we report high predictive accuracy for all three ontologies (Figure 2, model d). In fact, among the best performing and most specific predictions are those for Molecular Function (MF) GO terms “acyl-CoA dehydrogenase activity,” “transposase activity,” “organic acid∶sodium symporter activity” and its parent term “solute∶sodium symporter activity,” “penicillin binding” and its parent term “drug binding” (Figure S3 in Text S1).

### Model that includes paralogs provides more predictions with the same correctness

**Figure 3:**
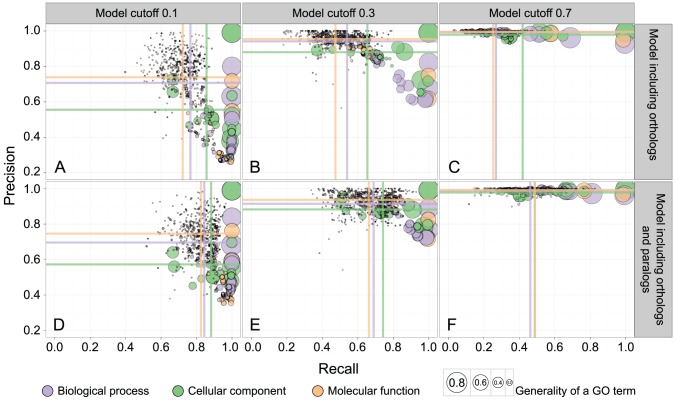
The relationship between Precision and Recall for GO terms, at various model stringency cut-offs. Predictions for each GO term are evaluated at one of three cut-offs: (A), (B), and (C) show results at cut-offs 0.1, 0.3, and 0.7 respectively, for the model including OMA cliques of orthologs and OMA inferred orthologs; (D), (E), and (F) show results at model cut-offs 0.1, 0.3, and 0.7 respectively, for the model including OMA cliques of orthologs, OMA inferred orthologs, and OMA inferred paralogs. Each disc represents one GO term; the colour denotes the ontology, and the area of the disc reflects the frequency of the GO term in the 07-02-2012 UniProt-GOA release. The coloured lines correspond to the mean values for the respective axes, for the respective ontology. The model made at least 50 predictions for each visualized GO term.

The AUPRC provides us with a view on predictive accuracy that values both the comprehensiveness of predicted annotations for a given GO term (Recall) and their correctness (Precision) across the entire range of model stringency cut-offs. To further explore the relationship between Precision and Recall at specific levels of model stringency, we chose three cut-offs—0.1 (permissive cut-off), 0.3 (medium cut-off), and 0.7 (stringent cut-off), for the two best models—the model including orthologs (corresponding to AUPRC values in Figure 2, model b) and the model including both orthologs and paralogs (corresponding to AUPRC values in Figure 2, model d). The combination of data and cut-offs resulted in six plots (Figure 3).

For any of the cut-offs, the mean Precision for GO terms between the two models is similar (Figure 3, horizontal lines between A and D; B and E; C and F). However, there is a difference for Recall, in particular for the more stringent cut-offs (Figure 3, vertical lines between B and E; C and F). It is this increase in Recall that increases AUPRC, as we observed before (Figure 2). For example, at the most stringent cut-off the model including only orthologs predicts annotations with 414 GO terms for at least 50 poorly characterized genes in the 998 genomes, while the model including both orthologs and paralogs predicts annotations with 573 GO terms for at least 50 genes.

To each unnannotated OMA clique, the model assigned a cut-off that indicates the probability of being annotated with a GO term. To have an interpretable measure of confidence for each prediction, we transformed this cut-off to the corresponding Precision (see the Materials and methods section). We then propagated the function of each OMA clique to the member genes and obtained the functional annotations, along with the estimates of Precision for each annotation.

**Figure 4:**
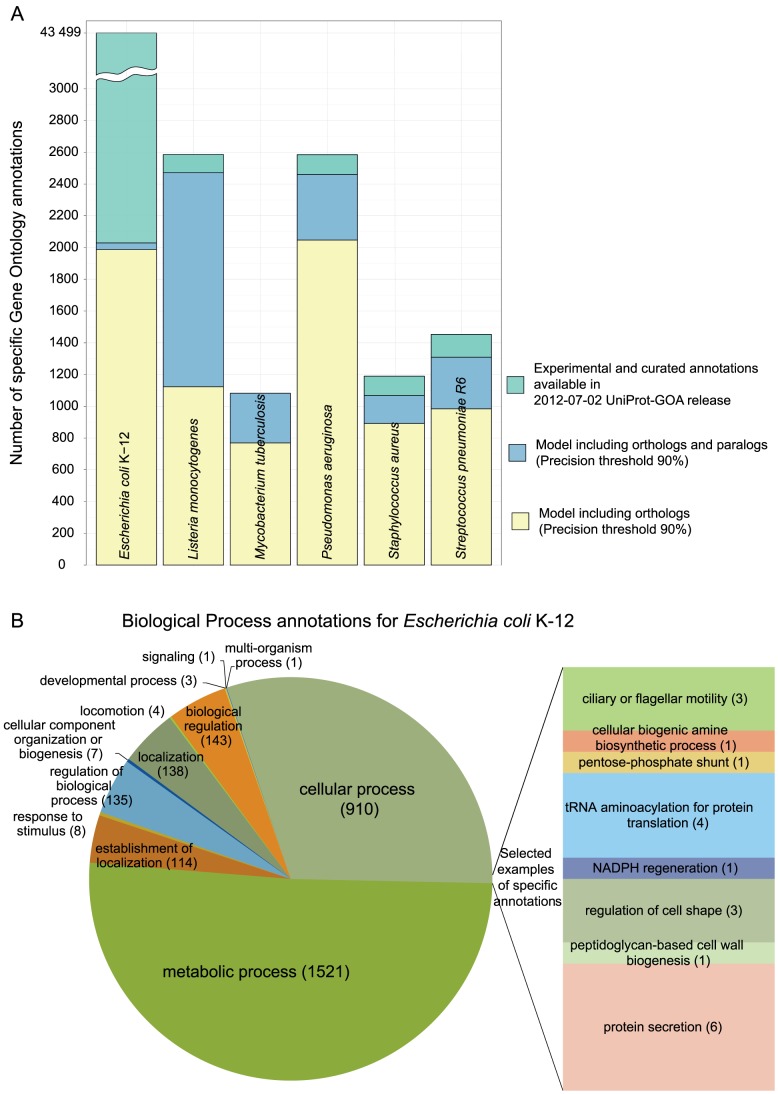
Existing and predicted annotations for the representative prokaryotes. A) The number of our model's predictions at Precision 90% compared to the available curated Gene Ontology (GO) annotations. Each bar summarizes the data for one prokaryote: *Escherichia coli* K-12, *Listeria monocytogenes* serotype 4b str. F2365, *Mycobacterium tuberculosis* H37Ra, *Pseudomonas aeruginosa* UCBPP-PA14, *Staphylococcus aureus subsp. aureus* NCTC 8325, and *Streptococcus pneumoniae* R6. For both our predictions and the available GO annotations we show GO term annotations that have Information Content higher than 3. The colour of the bar denotes the source of the annotations: yellow for the model that includes all inferred one-to-one, one-to-many, many-to-one, and many-to-many orthologs, blue for the model that includes both these orthologs and the paralogs, and green for the curated annotations available in the 07-02-2012 UniProt-GOA release. B) Biological Process (BP) annotations that the model including both orthologs and paralogs assigned to *E. coli* genes at Precision 90%. Apart from the most general terms in the BP ontology, we highlight some more specific annotations.

As a consequence of the increased Recall, the model that includes both orthologs and paralogs provides more annotations at the same Precision (Figure 4A). The increased Recall allows us to assign specific annotations at a very stringent threshold of 90% Precision. For example, we predict new annotations for *E. coli*, both using the most general, as well as many specific GO terms (Figure 4B, Figures S4 and S5 in Text S1).

### Experimental validation of the model's accuracy estimates

In the comparisons above, we obtained the best predictive performance for the model based on cliques of orthologs enhanced by both inferred orthologs and paralogs. We evaluated the ability of each model to generalize to novel data, the poorly characterized genes, with an out-of-bag method for testing predictive performance: we measured accuracy on a random subset of the annotated phyletic profiles left out when inferring the functional annotation model. This method was shown to give unbiased estimates of predictive performance [20].

**Figure 5:**
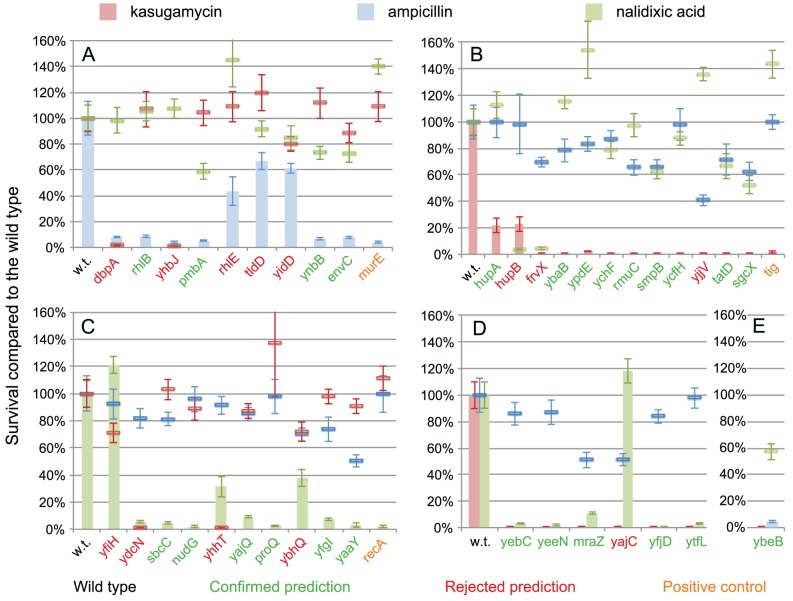
Experimental validation of predictions. A) Genes predicted to be annotated with “peptidoglycan-based cell wall biogenesis,” B) genes predicted to be annotated with “translation,” C) genes predicted to be annotated with “DNA damage response,” D) genes predicted to be annotated with both “translation” and “DNA damage response,” and E) a gene predicted to be annotated with both “translation” and “ peptidoglycan-based cell wall biogenesis.” The x-axis denotes the *Escherichia coli* knockout mutant. The y-axis represents the percentage of survival of the mutant strain normalized to the wild type. Coloured bars represent the survival when the antibiotic disrupts the biological process we predict for the genes; here, the correctly annotated mutants are expected to survive less than the wild type (w. t.). Coloured lines represent the survival when we predict no effect of the antibiotic on the survival rate; here, deletion mutants were expected not to differ from the wild type. Error bars show the variation in the results among the four replicates.

To validate how realistic are these out-of-bag performance estimates, we chose annotations for 38 genes in *Escherichia coli* K-12 having at least 60% expected Precision, for three GO terms that were straightforward to investigate experimentally using readily available antibiotics: “DNA damage response,” “translation,” and “peptidoglycan-based cell wall biogenesis.” The 38 *E. coli* strains, each with the deletion of one among the 38 selected genes, were grown in the presence of antibiotics that target the above Biological Processes: nalidixic acid (causes severe DNA damage, including double-strand breaks), kasugamycine (inhibitor of translation initiation), and ampicillin (inhibitor of cell wall synthesis) (Figure 5).

To each of the 38 genes the model assigned a Precision, as explained in the Materials and methods section. For example, Precision associated with the *E. coli* gene *yfgI* for “DNA damage response” was 62%; for “translation” and “peptidoglycan-based cell wall biogenesis” it was lower than 1%. We would therefore predict this gene to be involved in “DNA damage response” with a probability of being a false positive of 38% (100-62). For the other two GO terms the probability of being a false positive would be over 99%: the annotation model inferred that these are unlikely functions for this gene.

To experimentally evaluate a predicted annotation, we used the *E. coli* mutant deleted in the gene whose function we predicted. We compared the mutant to the *E. coli* wild type when grown in the presence of the antibiotic that inhibits the predicted function. If the gene is indeed involved in the predicted function, the survival of the mutant is expected to be lower than the survival of the wild type. For example, we predicted “DNA damage response” for the *E. coli yfgI* gene, so the corresponding mutant and the wild type were grown in the presence of DNA-damaging nalidixic acid; we expect the mutant to have lower survival than the wild type because its DNA repair capabilities are diminished.

We might predict a particular function, such as “DNA damage response,” for an important gene that is indirectly involved in many biological processes. Deleting such a gene may lower survival non-specifically and thus appear to validate our prediction. To control for this, we also grew each mutant in the presence of the two additional antibiotics. For the above example of the *yfgI* gene, if our prediction is correct, the survival of the mutant should not be different from the survival of the wild type when grown on kasugamycin or ampicillin.

Therefore, we considered a prediction confirmed only if both of the following criteria were satisfied: 1) the survival of a mutant was lower than 25% of the wild type when grown with the addition of the antibiotic inhibiting the process predicted by our model, and 2) the survival of the mutant was higher than 50% of the wild type when grown on the other two antibiotics.

For example, we predicted “DNA damage response” for the *E. coli yfgI* gene: when grown on DNA-damaging nalidixic acid, the *yfgI* mutant had 7% survival of the wild type, but when grown on kasugamycine or ampicilin, the survival was much higher: 98% and 74% of the wild type, respectively (Table S1). We therefore consider the prediction for the involvement of the *yfgI* gene in DNA repair processes confirmed: the *yfgI* mutant is sensitive to a DNA-damaging agent, while exhibiting wild type-like resistance to other stresses.

With these criteria, 25 out of 38 genes had confirmed predictions, which is equivalent to the experimental Precision of 66% (Figure 5). Since the selected genes had an expected Precision of 60%, the experiments show that the estimates of accuracy provided by the model are realistic. In fact, 14 of the 38 tested genes have Precision ≥85%. For these genes, the experiments have shown 11 out of 14 (79%) to be correct, approximately matching the expected precision of 85%.

Consequently, these estimates can be used to guide decisions when prioritizing genes for an in-depth experimental investigation of function in the wet lab.

### Examples of novel functions for *Escherichia coli* genes supported by literature evidence

In addition to the systematic experimental verification of novel annotations in three GO categories as described above, here we highlight individual predictions for which we found supporting evidence in the publicly available databases. This information was not available to the classifiers at the time when the models were constructed. The following examples are for *E. coli* K12, as this is by far the best-studied model prokaryote [21].

We predict genes *hypC* and *hybG* to have “nickel cation binding.” These genes had no GO terms assigned in the 07-02-2012 UniProt-GOA release (http://www.uniprot.org/uniprot/P0AAM3 and http://www.uniprot.org/uniprot/P0AAM7), and we therefore considered them unannotated. In the meantime, *hypC* was annotated with “metal ion binding” using experimental evidence: this is a parent GO term of our prediction. Moreover, when examining the literature, we found evidence that these two genes are involved in the biosynthesis of the [NiFe] cluster [22].

Another prediction is for *gltL*: we predicted it is annotated with “ATP-binding cassette (ABC) transporter complex.” In line with our predictions, PortEco, a portal that includes information from 14 different *E. coli* data resources [23], labels the gene as “ATP-binding component of ABC superfamily.” Note that more general electronic GO annotations were available for this gene, e.g. “ATP binding,” “ATPase activity,” and “ATP catabolic process” (http://www.uniprot.org/uniprot/P0AAG3).

A similar prediction of a more detailed function is for *ybgI*, where we predict GO terms from both BP and MF ontologies. This gene is known to be a conserved metal-binding protein [24], having an electronic GO annotation “metal ion binding”; we predict it is annotated with the BP GO term “Mo-molybdopterin cofactor metabolic process.” Based on the structure of the protein, Ladner *et al.* hypothesize this protein is a hydrolase-oxidase enzyme [24]; we predict this protein is annotated with the MF GO term “hydrolase activity, acting on acid anhydrides, in phosphorus-containing anhydrides.”

### Predictions available to browse or download from the GORBI website

**Figure 6:**
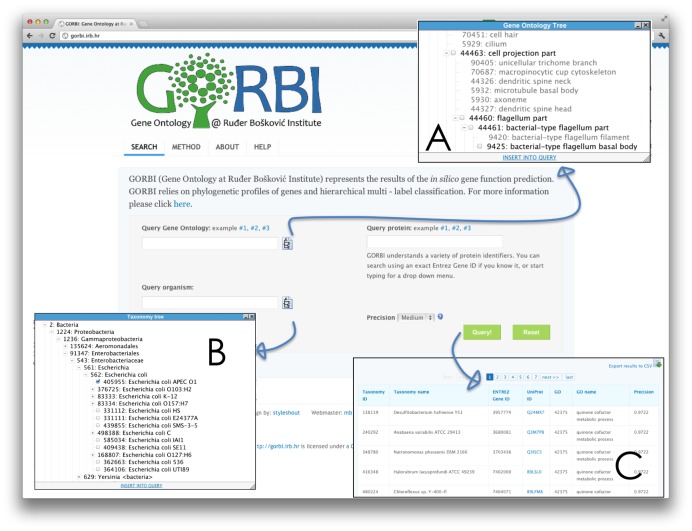
A screenshot of the GORBI web site. Predictions can be browsed in three ways, and any of their combination: 1) query using a Gene Ontology (GO) accession number (e.g. 6418 for “tRNA aminoacylation for protein translation”); alternatively, one can browse the “Gene Ontology DAG” (insert A) to find interesting GO terms, 2) query using an organism's NCBI taxonomy ID (e.g. 288681 for *Bacillus cereus* E33L); alternatively, one can browse the “Taxonomy tree” (insert B) to find the NCBI taxonomy IDs of interesting organisms, 3) query using protein identifiers: NCBI Gene ID, UniProtKB protein ID, RefSeq ID, UniRef ID, UniParc, or EMBL ID. The results (insert C) list NCBI taxonomy ID and the name of the organism, Entrez Gene ID, UniProt ID that links to the corresponding protein's page in the UniProt Knowledge Base, GO accession number, the name and the expected precision for the prediction.

Because we showed our functional annotation model gives realistic estimates of predictive accuracy, we made our predictions freely available in a Web site GORBI (http://gorbi.irb.hr/). Our predictions can be queried either using GO accession number, NCBI taxonomy ID, or gene/protein ID (Figure 6). For example, one can focus on more general or more specific GO terms, depending on their position in the “Gene Ontology DAG” (Figure 6, insert A). In addition, an experimenter can tune the search parameters to get a small number of high-confidence candidates, or a larger number of candidates that potentially have more false predictions, depending on the availability of annotations for the desired function and the available resources for experimental validation.

## Discussion

### Phyletic profiles in functional annotation

The intuition of phyletic profiling is that corresponding genes gained and lost together in different genomes are likely to share function: they could be involved in the same metabolic pathway, which is therefore incomplete without all the members in a genome [1]. Additionally, even if the two genes are parts of separate pathways and don't strictly require each other for function, they could both share a role beneficial for survival in a particular environment [25].

The standard way of finding corresponding genes in different genomes is via sequence homology: in addition to inferring function via homology, a phyletic profile allows to infer function based on the presence or absence of the corresponding genes in a range of organisms.

### Orthologs and paralogs in functional annotation

In functional annotation, we often differentiate between two subtypes of homologs, orthologs and paralogs [e.g., 26]. According to the standard model of genome evolution, paralogs—pairs of genes diverged through a duplication event—could obtain a new function [e.g., 27]. Conversely, orthologs are pairs of genes diverged through a speciation event and should be more likely to retain function; they are therefore expected to be more informative in functional annotation [15].

However, the exact quantification of the functional divergence in a pair of orthologs and a pair of paralogs is not fully resolved. It was observed that the search for homologs using the best bidirectional hit approach, without explicitly distinguishing orthologs from paralogs, produces a higher level of functional compactness via Gene Ontology (GO) terms [28] than is present in the ortholog databases Homologene [29] and OMA [30]. In addition, Studer and Robinson-Rechavi list scenarios where the standard model—predicting that paralogs diverge in function more than orthologs—is invalid; for example, cases where paralogs share function, and orthologs do not [31].

A recent large-scale study further challenged the veracity of the standard model: the authors compared mouse and human ortholog and paralog pairs and surprisingly found that paralogs tend to conserve function more than orthologs [32]. This finding caused a stir in the community—demonstrating the relevance of the topic—but was subsequently challenged in two publications [17], [33].

Nevertheless, a recent systematic survey showed that the divergence in function between paralogs is not as strong as the standard model would imply [17]. In addition, we know that homologs—orthologs and paralogs combined—are useful in functional annotation, especially when their sequence similarity is above the “twilight zone” [34]. Further, orthologs and paralogs share a common ancestor: paralogs, as well as orthologs, could carry functional information useful for annotation.

### Paralogs enrich phyletic profiles

In line with the recent research [17], our results show that the standard model, when viewed in the functional annotation context, tends to draw too strong of a line between orthologs and paralogs. When we enriched clique-only annotation models with additional orthologs and additional paralogs, we obtained a model that outperformed both the model that was enriched only with orthologs and the model that was enriched with refined homologs at different evolutionary distances (Figure S6 in Text S1). The improvement is most notable in the number of new annotations we were able to assign: while keeping the Precision at the same high level, our best model increases Recall (Figure 3), and consequently gives us more predictions at the same level of correctness (Figure 4A).

**Figure 7:**
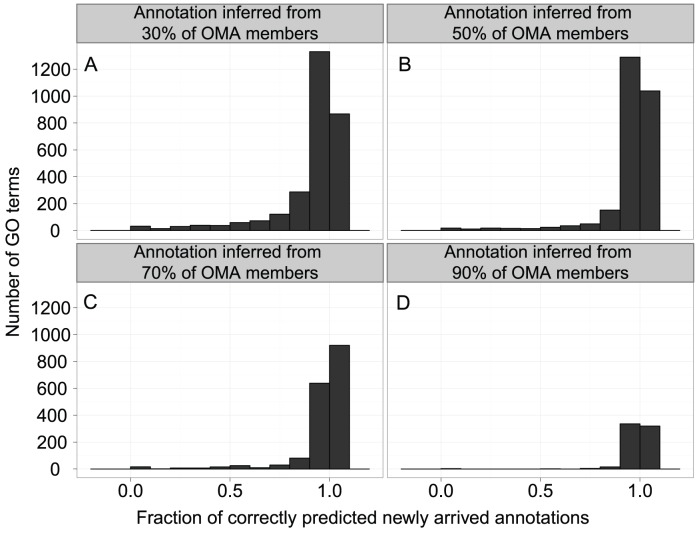
Functional coherence of GO annotations. Each panel presents the results of evaluation for the annotations inferred from A) 30%, B) 50%, C) 70%, and D) 90% of OMA members. We propagated the annotations available for the OMA group members in the 2008-01-16 UniProt-GOA release to unannotated group members, and evaluated the predictions with the newly arrived annotations in the more recent 17-10-2011 UniProt-GOA release.

Even so, our results do not contradict the standard model in two major points: 1) cliques of orthologs—groups where all genes are connected with orthologous relations—are indeed functionally very similar (Figure 7), and 2) our results support the current widespread annotation efforts that use homology: even when we disregard the orthology/paralogy relationships to enhance cliques, we obtain high predictive accuracy (Figure S6 in Text S1).

### Orthologs-only model outperforms paralogs-only model

The OMA algorithm infers paralogs (i.e., non-orthologs) among genes linked as the best bidirectional hits in the respective genomes: a witness to non-orthology breaks the link between two genes [35]. Because only one witness is enough to break the orthologous relationship, the OMA algorithm produces orthologous groups with high specificity [36]. As a trade-off, the set of inferred paralogs might contain pairs whose orthologous link was erroneously broken; the probability for this to happen increases with the addition of new genomes (A. Altenhoff, personal communication). Therefore, our set of paralogs might contain orthologs that were misclassified as paralogs.

Even so, when we enriched clique-only annotation models with the inferred paralogs, predictive accuracy increased *less* than when we enriched clique-only annotation models with the missing orthologs (Figure 2, models c and b, respectively). We obtained these results despite enriching with a larger number of paralogous pairs than orthologous pairs: it is not the number of added pairs that improves the predictive accuracy, but the genome they are located in.

### Experimental validation shows that the model's performance estimate is realistic

An important output of any computational annotation model is an estimate of confidence for the annotations: it can subsequently be used to guide decisions about experimental validation. In fact, one project that provides a framework for the exchange of information between the computational and experimental communities is COMBREX [37], [38]. To meaningfully contribute to growing resources such as COMBREX, we wanted to evaluate whether our annotation model provides realistic estimates of confidence for the individual annotations.

Probing growth profiles of knockout *E. coli* mutants with sub-lethal concentrations of antibiotics is an established method of functional annotation [39], [40]. Here, we experimentally validate whether a gene is involved in the predicted Biological Process by growing the respective knockout *E. coli* mutant in a medium containing the antibiotic that targets the Biological Process we predicted. The experimental results support the estimates of Precision obtained from a cross-validation procedure, serving as a proof of principle that our phyletic profiling-based model is useful when searching for novel functions of poorly annotated genes in a microbiology lab.

Our annotation model assigns GO terms from across the GO hierarchy, for both general and specific terms. Overall, more general terms tend to have a higher cross-validation Area Under the Precision-Recall Curve (AUPRC) (Figure S3 in Text S1) and consequently the annotations assigned with these terms are more likely to be correct.

The AUPRC scores such as the one we use serve as a test of the internal consistency of the model. On the one hand, the model captures the similarities of the phyletic profiles of the OMA cliques (and the enriched OMA cliques) of orthologs; on the other hand, the model captures the GO terms assigned to the OMA cliques of orthologs. For a given GO term, the AUPRC scores will be low if the phyletic profiles' features cannot be used to transfer the function between the profiles. Thus, we make no prior assumptions whether a GO term at a certain level of specificity can be transferred across profiles, but rather infer this from the data itself in a systematic manner.

An experimenter can focus on more general or more specific GO terms depending on the trade-off of reported Precision and the cost/time required of experiments; when using the GORBI Web site, GO terms can be selected depending on their position in the Gene Ontology hierarchy (Figure 6, insert A).

To facilitate the use of the generated computational annotations, we provide them in a Web site GORBI (http://gorbi.irb.hr/) where each prediction is accompanied by the annotation model's estimate of confidence.

### Conclusions

We contribute a solution for computational annotation of genes that utilizes a distinction between two types of homologs—orthologs and paralogs—to yield an innovative annotation model: phyletic profiles derived from cliques of orthologs enriched with both orthologs and paralogs have shown the best predictive accuracy. Our results are in line with related recent research: while it is generally accepted that pairs of orthologs have a lower rate of functional divergence, the divergence in paralogous pairs is not as strong as the standard model would imply [17].

In addition, we performed validation experiments in knockout mutants of *E. coli*, showing that our annotation model reports realistic measures of predictive performance. The agreement with the experimental results implies that our functional annotations—and the corresponding confidence estimates—can be used to narrow the search space for potential function candidates and thereby help to bridge the widening gap between the sequenced and characterized proteins.

For successful annotation of newly sequenced proteins, we need contributions from both the computational community—a large number of credible annotations—and the experimental community—validating the most interesting computational annotations. In turn, the validated findings from the wet-lab can be fed into the computational annotation pipelines, helping to propel a virtuous circle that increases the number of experimentally annotated genes.

Our research aims to contribute to the understanding of the deluge of data we face, whether from complete microbial genomes for which we provide annotation predictions (http://gorbi.irb.hr/), or from the metagenomics projects, in particular the emerging human microbiomes, to which we can apply our annotation model.

## Materials and Methods

### Annotation data

We downloaded all annotation data from the FTP site of the UniProt-GOA database [41].

We used the Gene Ontology (GO) vocabulary for functional annotation [28]. We included all annotations assigned by a curator (evidence codes EXP, IMP, IGI, IPI, IEP, IDA, ISS, RCA, IC, NAS, TAS), and from the non-curated annotations (evidence code IEA), we included those inferred from UniProtKB keywords, UniProt Subcellular Location terms, Enzyme Commission numbers, and InterPro (reference codes GO_REF:0000004, GO_REF:0000023, GO_REF:0000003, and GO_REF:0000002, respectively). Despite not being curated, a recent report showed these electronic annotations are of high quality, in particular for the only analysed Prokaryote, *E. coli* [42] and Figure S7 in Text S1

We express the specificity (opposite of generality) of a GO term GO_i_ with respect to its Information Content:
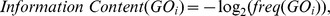
where freq(GO_i_) is the frequency of GO_i_ among all annotations for the twelve Reference genomes [43].

### The OMA algorithm and the OMA database

The OMA algorithm is a graph-based method of orthology inference [35]. Roth *et al.* provide full details of the algorithm, and we summarize the main points relevant to our work. The algorithm starts with an all-against-all sequence alignment: proteins from two species are connected if they are best bidirectional hits, within a confidence interval, in the compared species. The connections between a pair of proteins are broken when one of them is the best bidirectional hit with one of the proteins in a connected pair in some third species, and the other is the best bidirectional hit with the second protein in the same pair; the broken pairs are inferred paralogs. The remaining connections are inferred orthologs. Finally, OMA cliques of orthologs are sub-graphs where all proteins are connected by orthologous relationships (Figure 1).

In this work, we only use OMA cliques that group at least 10 members.

The OMA algorithm is available as a stand-alone version; the results can also be browsed on the OMA web site [44].

### Annotating OMA cliques of orthologs

Because one essential component of our work is annotating OMA cliques of orthologs based on the proteins they contain, we first checked whether OMA cliques contain proteins with the same function. First, unannotated OMA members were labelled with the GO terms of annotated OMA members at four thresholds: if 30, 50, 70, or 90% of OMA members have the respective function. To assign these labels, we used only annotations available in the 16-01-2008 UniProt-GOA release.

Next, we checked the annotations in the more recent 17-10-2011 UniProt-GOA release. For each unannotated protein, we consider the labelled function to be *confirmed* if the protein holds the respective annotation in the more recent release; we consider the labelled function to be *rejected* if the protein holds the same annotation alongside a ‘NOT’ qualifier (explicit rejection) or a new annotation that is not the propagated one (implicit rejection). To make a more robust measure, we summarize the confirmed and rejected annotations for each GO term. We named this measure ‘Coherence of a GO term.’ More formally,
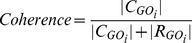
where 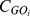 is the set of confirmed annotations associated with term GO_i_ and 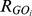 is the set of rejected annotations associated with term GO_i_. We account for the definition of the GO: the assignment of any GO annotation assumes the assignment of all the GO parent terms.

This is a conservative estimate of Coherence: we consider as rejected an annotation that might not have been added to the database yet. Annotations are continuously being added to UniProt-GOA database, and the annotation update interval for a gene can be as long as 12 years [42]. To compensate for this bias, we evaluated coherence on a three-year interval, as most genes in *E. coli* are updated within that time frame.

For each GO term, the functional coherence depends on the imposed annotation threshold (Figure 7): when a larger fraction of OMA members in 2008 supported the GO annotation, we found more newly annotated proteins that support this propagated GO annotation in 2011. The drawback of the increasing threshold was a smaller number of GO terms that can be used in annotation and consequently a smaller number of annotated OMA groups used in training the annotation model. We chose the threshold of 50% as a compromise: for most GO terms, the newly annotated proteins are in accordance with the propagated functions—fraction of correctly predicted newly arrived annotations is greater than 0.9—and we are left with enough specific GO terms for functional annotation (Figure 7, panel C): 422 GO terms from the Biological Process ontology, 48 GO terms from the Cellular Component ontology, and 264 GO terms from the Molecular Function ontology. We use the 50% threshold throughout this work.

### Phyletic profiles

The phyletic profile of an OMA clique of orthologs is encoded as a vector of binary values. The vector's length is 998 items—the number of prokaryotic genomes included in our work. Each position in the vector indicates the presence or absence of an OMA clique member in the respective genome. There are 64052 annotated and unannotated OMA phyletic profiles in our dataset (Figure 1).

We enriched the phyletic profiles, first by connecting the missing orthologs to OMA clique members (Figure 1, full lines), and second by connecting the paralogs (Figure 1, dashed lines) to OMA clique members. Orthologs include one-to-one orthologs, one-to-many orthologs, many-to-one orthologs, and many-to-many orthologs.

### Machine learning algorithms

The Clus-HMC [7] algorithm builds decision trees for hierarchical multi-label classification (HMC). In contrast to ordinary classification trees [45], which can be used for single-label annotation, Clus-HMC is able to deal with multiple, hierarchically organized class labels, such as terms from the Gene Ontology. It builds decision trees for HMC by extending the standard decision tree learning algorithm: It splits the training data into subsets based on attribute values, in order to minimize the weighted sum of variances for all class labels within the subsets resulting after the split [7].

In this weighted sum, a parameter w_0_ can be used to place more weight on either the more specific, or the more general GO terms. The default value of this parameter is 0.75, which places more weight on more general terms. Changing the default value of the w_0_ parameter to place more weight on the specific terms will favour them, possibly trading off the accuracy of the more general terms for a gain in accuracy of the more specific terms. To test for possible gain, we experimented with different values of the w_0_ parameter to place higher weight on either the more general GO terms (default value, w_0_ = 0.75; w_0_ = 0.5) or on the more specific GO terms (w_0_ = 1/0.75 = 1.33; w_0_ = 1.75; w_0_ = 2.0; w_0_ = 3.0). Clus-HMC-Ens proved to be robust to the value of the w_0_ parameter (Figure S8 in Text S1): we did not record a significant change in the AUPRC values (p-value was not lower than 0.28 in the five tested values of the w_0_ parameter, Wilcoxon signed-rank test), and we therefore used the default value in all our computational experiments.

In addition, the hierarchy of class labels introduces dependencies between the classes: Clus-HMC is aware of the hierarchical relationships between the multiple labels and uses this information to improve predictive performance.

The Clus-HMC algorithm was extended to an ensemble setting (Clus-HMC-Ens) [18], where a forest of decision trees for HMC is learned: The predictions of the individual trees are combined to obtain the overall prediction of the ensemble. Clus-HMC-Ens implements, among other methods, the Random Forest (RF) ensemble [19] approach, where the individual trees are constructed by using a randomized version of Clus-HMC. Each tree is constructed from a different sample of the training dataset: The bagging (Bootstrap aggregating) methodology of resampling the dataset [46] is used to construct the different samples. One bootstrap sample consists of the same number of examples as the original dataset, but they are randomly drawn *with replacement*; consequently a bootstrap sample contains about two thirds of unique examples. A model—Clus-HMC decision tree—is produced from each of the bootstrap samples.

When estimating the classification error, out-of-bag estimates are calculated. The examples that were *omitted* from the bootstrap sample—one third of the original dataset—are used in calculating Precision, Recall, and Area Under the Precision-Recall Curve (AUPRC). The estimates are based on the random sample, and the measures are therefore unbiased. To check whether adding paralogs improves the functional annotation model regardless of the machine learning algorithm used, we inferred functional annotation models with the standard approach used in phyletic profiling: transfer of function via pairwise distance measures between phyletic profiles, as implemented in a kNN classifier (Figure S9 in Text S1). The conclusions presented above do not change: the model that includes both orthologs and paralogs outperforms the model that includes only orthologs. Because Clus-HMC-Ens outperforms kNN in computational efficiency and predictive accuracy, we used Clus-HMC-Ens throughout this work.

### Evaluating the functional annotation models

We compare models of functional annotation using Precision-Recall curves: in the Precision-Recall space, Recall is on the x-axis, and Precision is on the y-axis. Traditionally, Precision and Recall are defined for binary classification: an instance either has or does not have the label; in our case, each OMA clique either has or does not have a GO annotation. Precision and Recall are defined for each GO term:
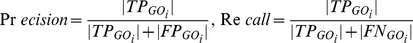
where 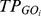 is the number of correctly predicted true annotations (“True Positives”), 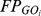 is the number of incorrectly predicted true annotations (“False Positives”), and 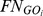 is the number of missed true annotations (“False Negatives”).

Precision stands for the fraction of correctly predicted examples out of all the predictions, and Recall stands for the fraction of correctly predicted examples out of all known to be true.

Here, we are dealing with a multi-class problem: each OMA clique can be annotated with multiple GO terms. The classifier we are using is adapted for such a problem and assigns a *probability* that each OMA group is assigned each of the GO terms. By varying a cut-off for the probability form 1.0 to 0.0, we are relaxing the stringency of the predictions: an increasing number of OMA groups are assigned an increasing number of GO terms. Fixing this cut-off at the three values and calculating Precision and Recall for each GO term created visualizations in Figure 3.

The probabilities allow us to have a ranking of GO annotation predictions for OMA cliques and proteins therein. In addition to the ranking, we wanted to have an intuition for the number of candidates we need to experimentally examine in order to get confirmed annotations. Therefore, we translated the probabilities to Precision for each GO term. Similarly as above, we varied the cut-off for the probability, and calculated the corresponding Precision for each GO term at each probability cut-off: out of all the OMA clique annotations that pass the threshold, we counted the number of true positives, and the number of false positives.

To compare models in Figure 2, we used a single measure of performance that combines Precision and Recall: Area Under the Precision-Recall Curve (AUPRC). To calculate AUPRC, we first varied the probability cut-off from 1.0 to 0.0 and obtained the Precision-Recall curve. We then calculated the area that is enclosed between the Recall axis and the curve. The closer AUPRC is to 1.0, the better the model.

### Bacterial strains, growth conditions, and antibiotic treatments

All deletion mutants used herein were derived from wild-type sequenced *Escherichia coli* MG1655 by P1 transduction. P1 phage was grown on a series of Keio collection deletion mutants listed in Table S1. Successfully transduced mutants were selected on LB plates supplemented with kanamycine.

Bacteria were grown in LB broth at 37°C, to the exponential phase (OD600 = 0.2–0.3). Viable cell counts were estimated by plating serial dilutions on LB plates, as well as LB plates supplemented with 400 ug/mL kasugamycine (inhibitor of translation initiation), 4 ug/mL nalidixic acid (causes severe DNA damage, including double strand breaks), and 3 ug/mL ampicillin (inhibitor of cell wall synthesis). Plates were incubated overnight at 37°C. The concentrations of antibiotics used in this study were selected as the concentrations that lead to ∼10% survival of the wild type *E.coli*.

### Sources of data and software

1. The orthology and paralogy data from the OMA database, May 2011 version was kindly provided by A. Altenhoff.
2. The cross-references for the various gene/protein identifiers (UniProt, GenBank, Entrez GeneID) were downloaded from the NCBI FTP site [http://www.ncbi.nlm.nih.gov/Ftp/].
3. GO annotations were downloaded from the UniProt-GOA FTP site. We used the 2008-01-16 and the 2011-10-17 UniProt-GOA releases to evaluate the consistency of OMA group annotations, 2011-10-17 UniProt-GOA release to create all the annotation models, and 07-02-2012 UniProt-GOA release to estimate the frequency of occurrence of a GO term in the UniProt-GOA database [http://www.ebi.ac.uk/GOA/].
4. The GO definition was downloaded from the GO FTP site [http://www.geneontology.org/GO.downloads.ftp.cvs.shtml].
5. Final dataset files in ARFF format, as given to the Clus-HMC-Ens algorithm (Datasets S1 and S2).
6. The Clus-HMC-Ens algorithm is available for download as part of the predictive clustering framework Clus [http://www.cs.kuleuven.be/~dtai/clus/].

## Supporting Information

Dataset S1 The *training* dataset files in ARFF format, as given to the Clus-HMC-Ens algorithm, for the Biological Process, Cellular Component, and Molecular Function ontology.
(ZIP)

Dataset S2 The *unlabelled* dataset files in ARFF format, as given to the Clus-HMC-Ens algorithm, for the Biological Process, Cellular Component, and Molecular Function ontology.
(ZIP)

Dataset S3 The settings files as given to the Clus-HMC-Ens algorithm.
(ZIP)

Table S1 Results of the experimental assays.
(XLSX)

Text S1 Supplementary figures.
(PDF)
