# Supplementary material for: Phyletic Profiling with Cliques of Orthologs Is Enhanced by Signatures of Paralogy Relationships
Source: PLoS Comput Biol. 2013 Jan 3;9(1):e1002852. doi: 10.1371/journal.pcbi.1002852 (PMC3536626; doi:10.1371/journal.pcbi.1002852)
Supplement: Text S1 — Supplementary figures. (PDF) [file pcbi.1002852.s005.pdf]

Text 1. Supplementary figures

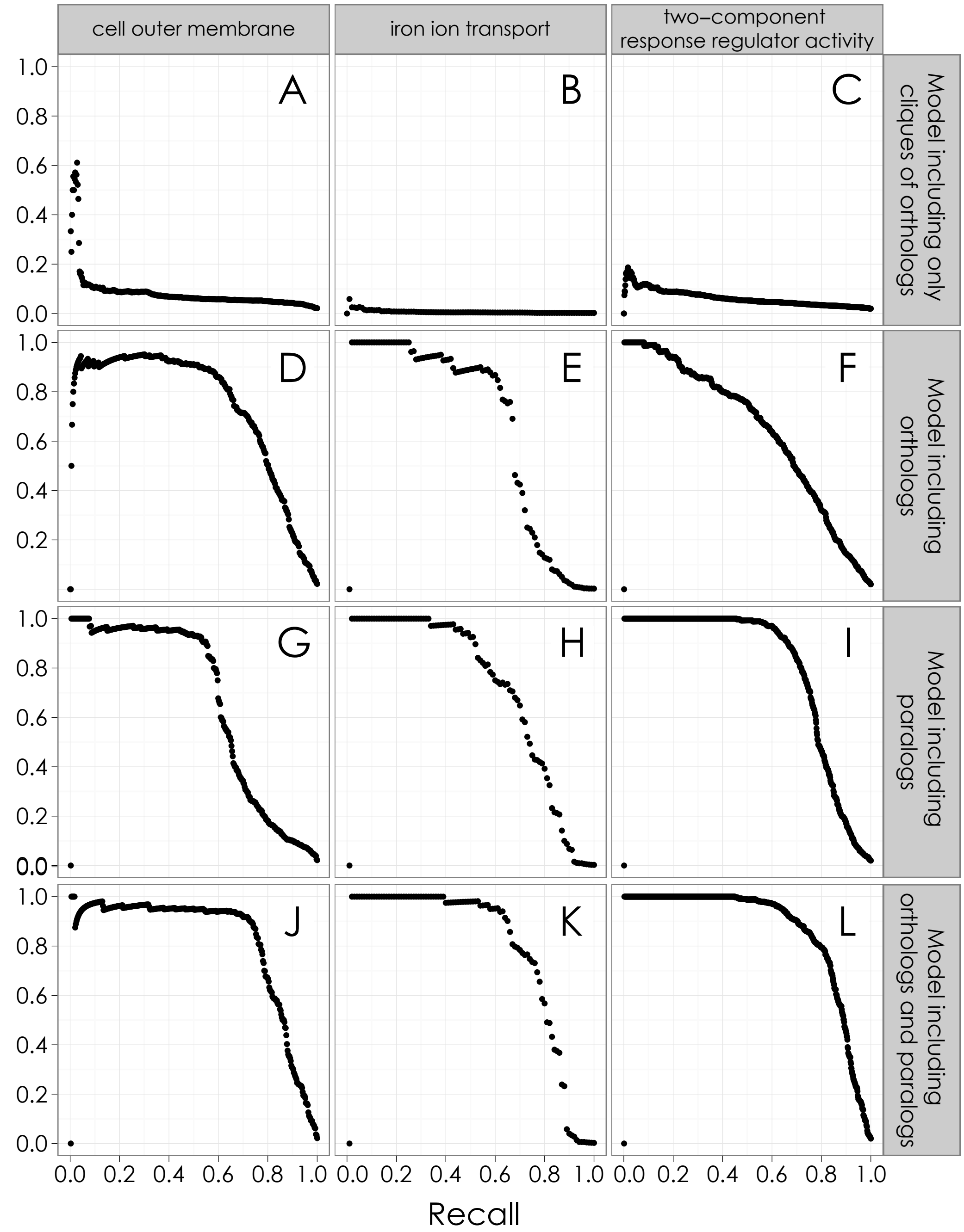

Figure S1: Examples of Precision-Recall curves for three Gene Ontology (GO) terms for the four annotation models. Each panel shows the Precision-Recall curve for the GO term denoted in the top title panel, obtained in the annotation model denoted in the right title panel. The x-axis represents Recall, and the y-axis represents Precision. The plots were created by increasing the cut-off for the model's annotation probability: each point in the plot represents the cumulative Precision and Recall at each step of varying the cut-off.

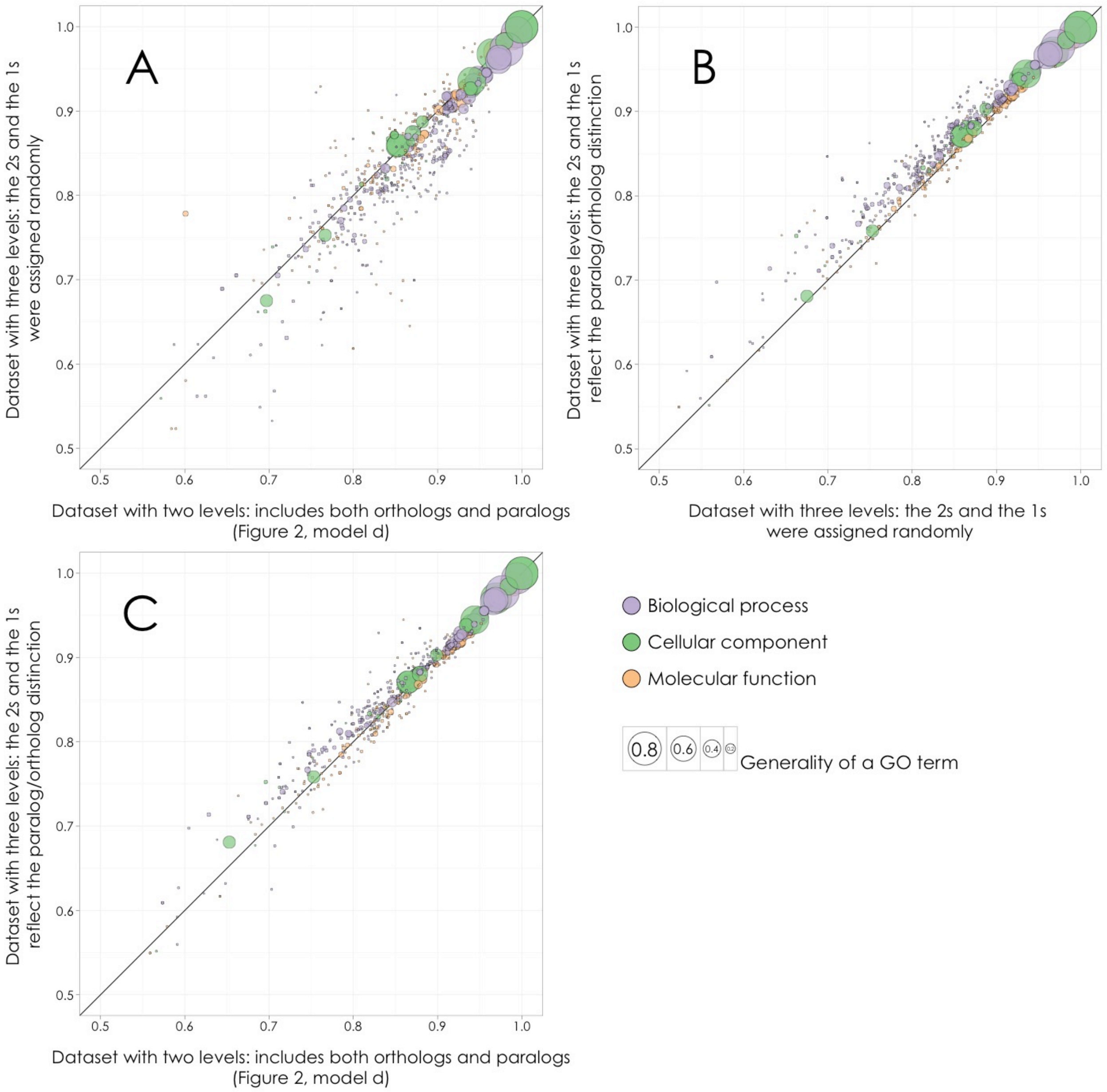

Figure S2: Classifier accuracy and the distinction between the orthologs and the paralogs. To check whether giving more weight to orthologous relations improves the predictions, we accounted for orthologous relations separately from the paralogous relations—in addition to the binary phyletic profiles, we have also tested a representation of the data with three levels: presence of an OMA clique member or another ortholog (2), presence of a paralog (1), or absence of any of these (0).

In the visualization, each disc represents one GO term; its colour represents the ontology, while the area of the disc is proportional to the frequency of the GO term among all annotations available in 07-02-2012 UniProt-GOA release. The x and the y-axes denote the Area Under the Precision-Recall Curve (AUPRC) obtained in the experiments denoted on the respective axis and described below.

(A) Datasets with more levels—in this case 3 instead of the original 2—represent a challenge for machine learning algorithms: the statistical support for smaller GO categories diminishes because of fewer data points per level. We performed a computational experiment that confirms this: after we had included three categories that did not reflect the paralog/ortholog distinction (i.e., the 2s and the 1s were assigned randomly), the AUPRC scores dropped compared to the binary dataset (Figure 2, model d), even though no information was removed (Wilcoxon signed-rank test p=0.0025).

(B) An experiment where the 1s and the 2s did reflect the paralog/ortholog distinction. The AUPRC scores showed a modest but notable increase when compared to the negative control in panel A, consisting of randomly assigned 1s and 2s (Wilcoxon signed-rank test p=1.6e-05).

(C) In the third experiment, we examined the practical usefulness of the orthology/paralogy distinction in our machine learning framework. Here, the dataset with three levels was compared to the original binary dataset: we did not observe a significant increase in the AUPRC scores (Wilcoxon signed-rank test p=0.2401).

Taken together, these results imply: i) that there is useful information that can be recovered when treating the orthologs and the paralogs separately, but also ii) that in our machine learning setup most of this gain is cancelled out by the increased difficulty of learning due to an increased number of levels (2 compared to 3).

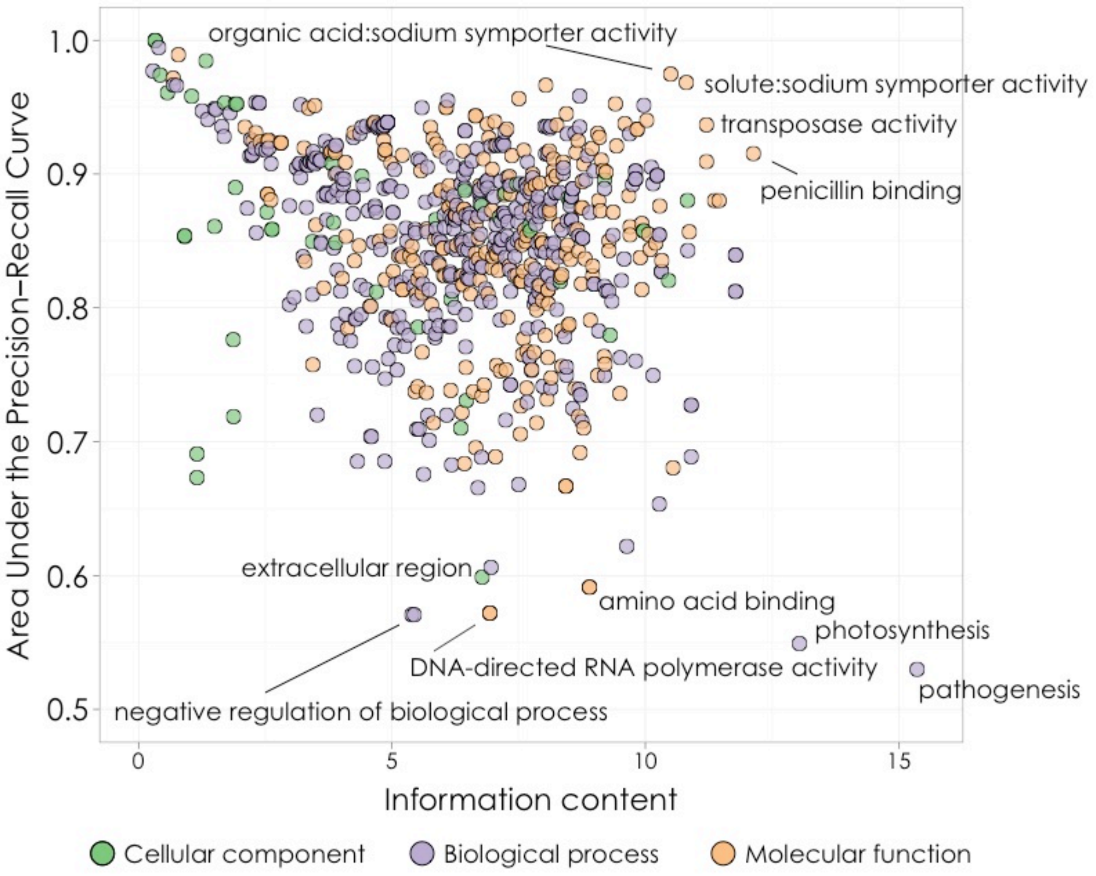

Figure S3: A comparison of Area Under the Precision-Recall Curve (AUPRC) and Information Content (IC) for the functional annotation model that includes OMA cliques of orthologs, OMA inferred orthologs, and OMA inferred paralogs. Each point represents one Gene Ontology term and its colour denotes the ontology. The IC is calculated as the negative logarithm of the frequency of the GO term in the 07-02-2012 UniProt-GOA release.

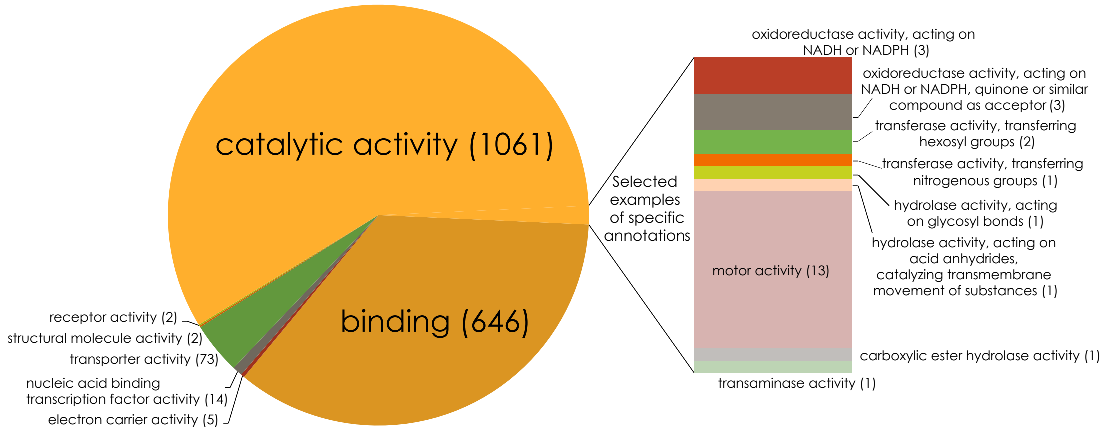

Figure S4: Molecular Function (MF) annotations that the model including both orthologs and paralogs assigned to *E. coli* genes at Precision 90%. Apart from the most general terms in the MF ontology, we highlight some more specific annotations.

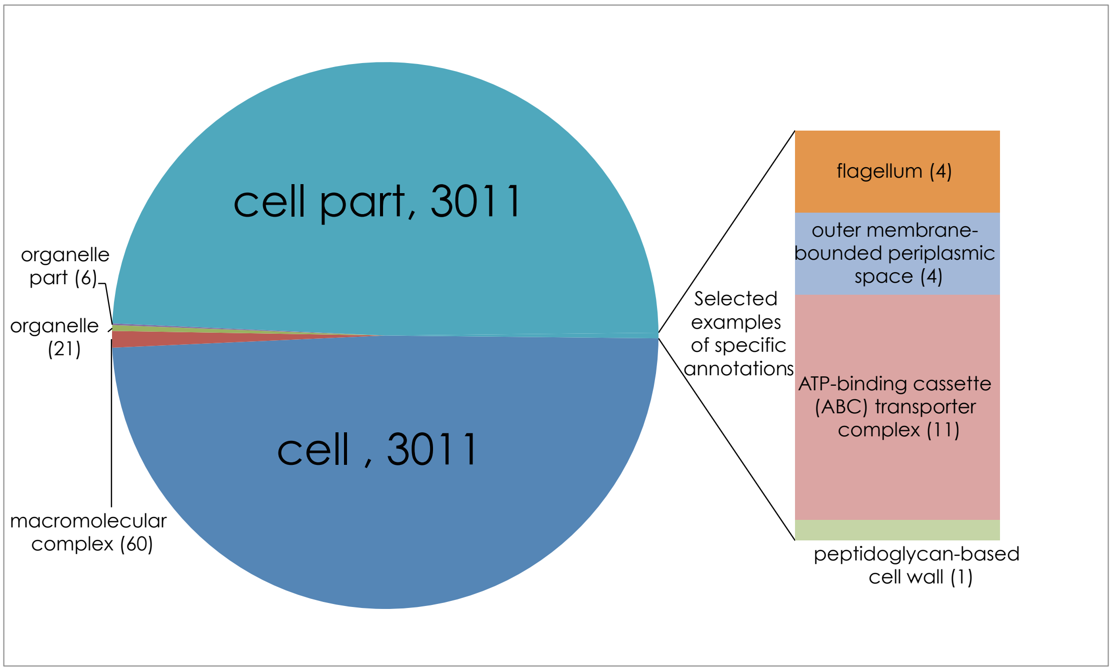

Figure S5: Cellular Component (CC) annotations that the model including both orthologs and paralogs assigned to *E. coli* genes at Precision 90%. Apart from the most general terms in the CC ontology, we highlight some more specific annotations.

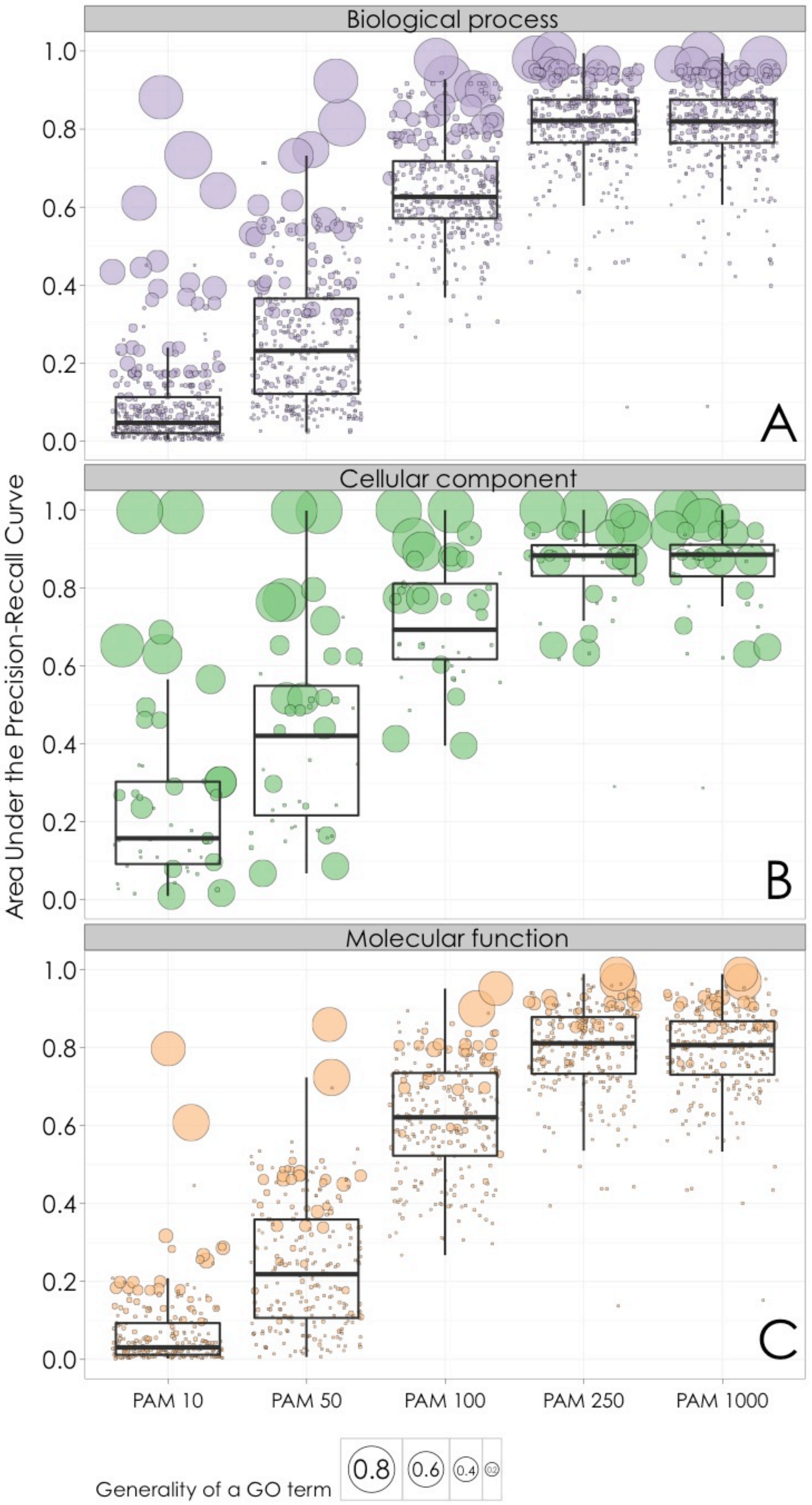
Biological process
Cellular component
Molecular function
Area Under the Precision-Recall Curve
1.0
0.8
0.6
0.4
0.2
0.0
A
B
C
PAM 10
PAM 50
PAM 100
PAM 250
PAM 1000
Generality of a GO term
0.8
0.6
0.4
0.2

Figure S6 (previous page). Predictive performance of the annotation models that account only for the evolutionary distance between refined homologs inferred by the OMA algorithm, for the three Gene Ontologies: A) Biological Process, B) Cellular Component, and C) Molecular Function. The x-axis represents the models: each model includes refined homologs that are closer than the denoted PAM distance. These homologs and their corresponding pairwise evolutionary distances were inferred by the OMA algorithm (Roth, Gonnet, & Dessimoz, 2008): all-against-all local sequence alignments were refined using two empirical criteria: first, only homologs with an E-value of roughly 10^{-14} were considered significant and second, only alignments where the shorter sequence is at least 61% length of the longer sequence are considered. The y-axis represents the Area Under the Precision Recall Curve (AUPRC). Each disc represents one GO term; its colour represents the ontology, while the area of the disc is proportional to the frequency of the GO term among all annotations available in 07-02-2012 UniProt-GOA release. Each boxplot summarizes AUPRC for the dataset indicated on the x-axis. Lower, mid, and upper horizontal lines denote the first quartile, median and the third quartile, respectively; vertical lines reach 1.5 interquartile range from the respective quartile or the extreme value, whichever is closer.

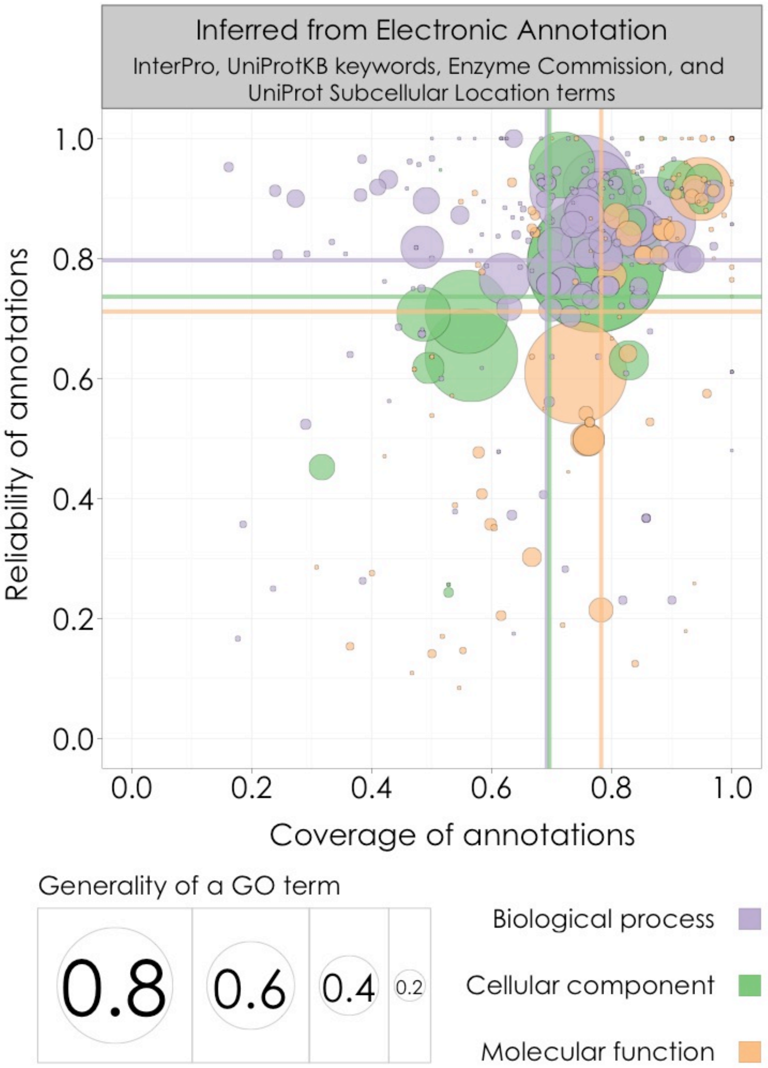

Figure S7: The quality of the 21-01-2009 UniProt-GOA release, evaluated by the 07-02-2012 UniProt-GOA release for electronic annotations assigned to *Escherichia coli*. A scatterplot of coverage compared to the reliability for the GO terms of the three ontologies: Biological Process, Cellular Component, and Molecular Function. The area of the disc reflects the frequency of the GO term in the 16-01-2008 UniProt-GOA release. The coloured lines correspond to the mean values for the respective axes. To be visualized in this plot, a GO term needs to have assigned at least 10 electronic annotations in the 21-01-2009 UniProt-GOA release and at least 10 experimental annotations in the 07-02-2012 UniProt-GOA release. The methodology used to obtain the data presented in this figure is described in (Škunca, Altenhoff, & Dessimoz, 2012). Note that this figure shows the analysis with more recent data than what is published by Škunca *et al*.

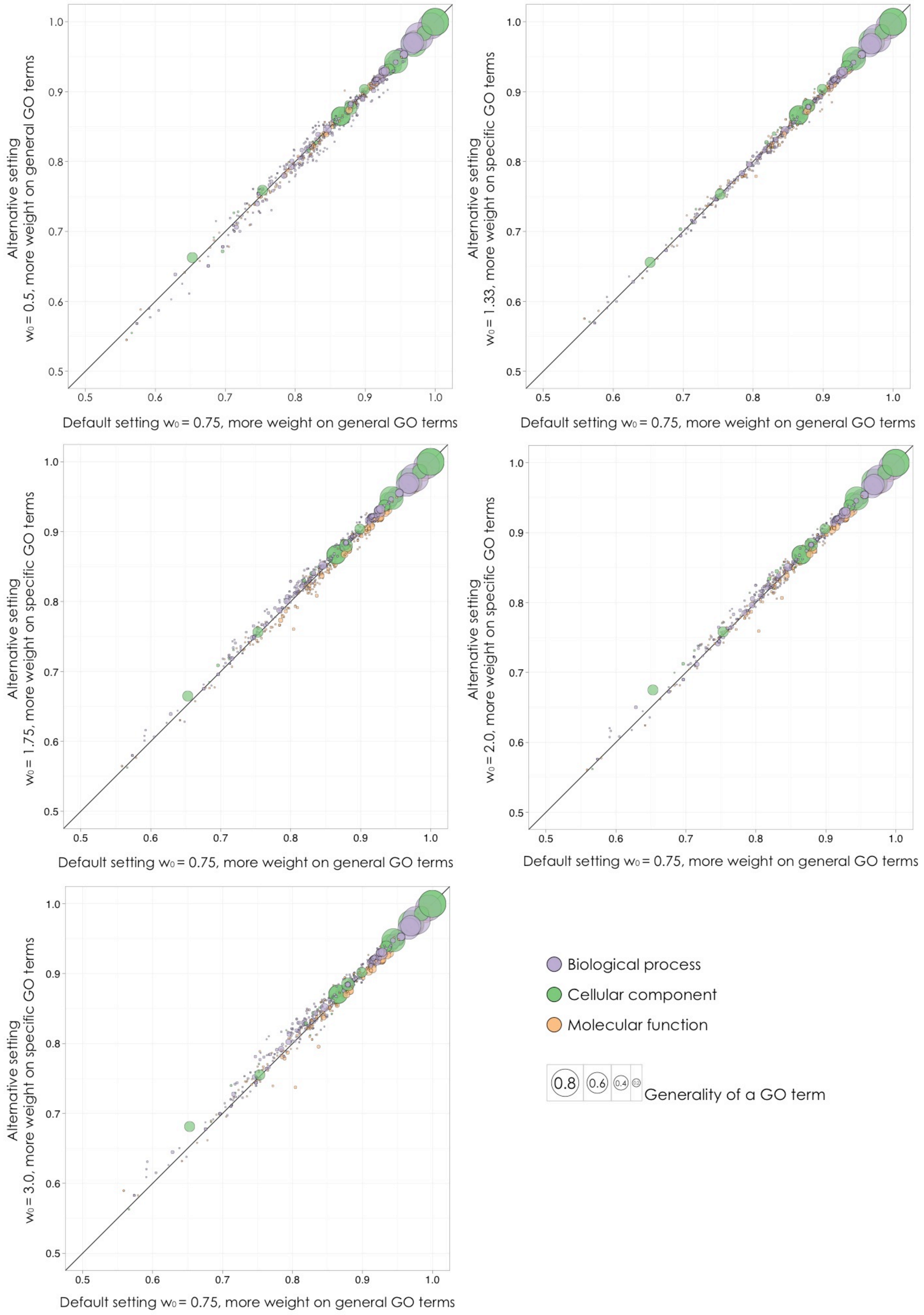

Figure S8: Testing the weight parameter of the Clus-HMC-Ens algorithm. The x-axis denotes the Area Under the Precision-Recall Curve (AUPRC) obtained in the experiment with the default value of the weight parameter w_0, and the y-axis denotes the AUPRC obtained in the experiment with the alternative value of the weight parameter w_0 denoted in the axis title. Each disc represents one GO term; its colour represents the ontology, while the area of the disc is proportional to the frequency of the GO term among all annotations available in 07-02-2012 UniProt-GOA release.

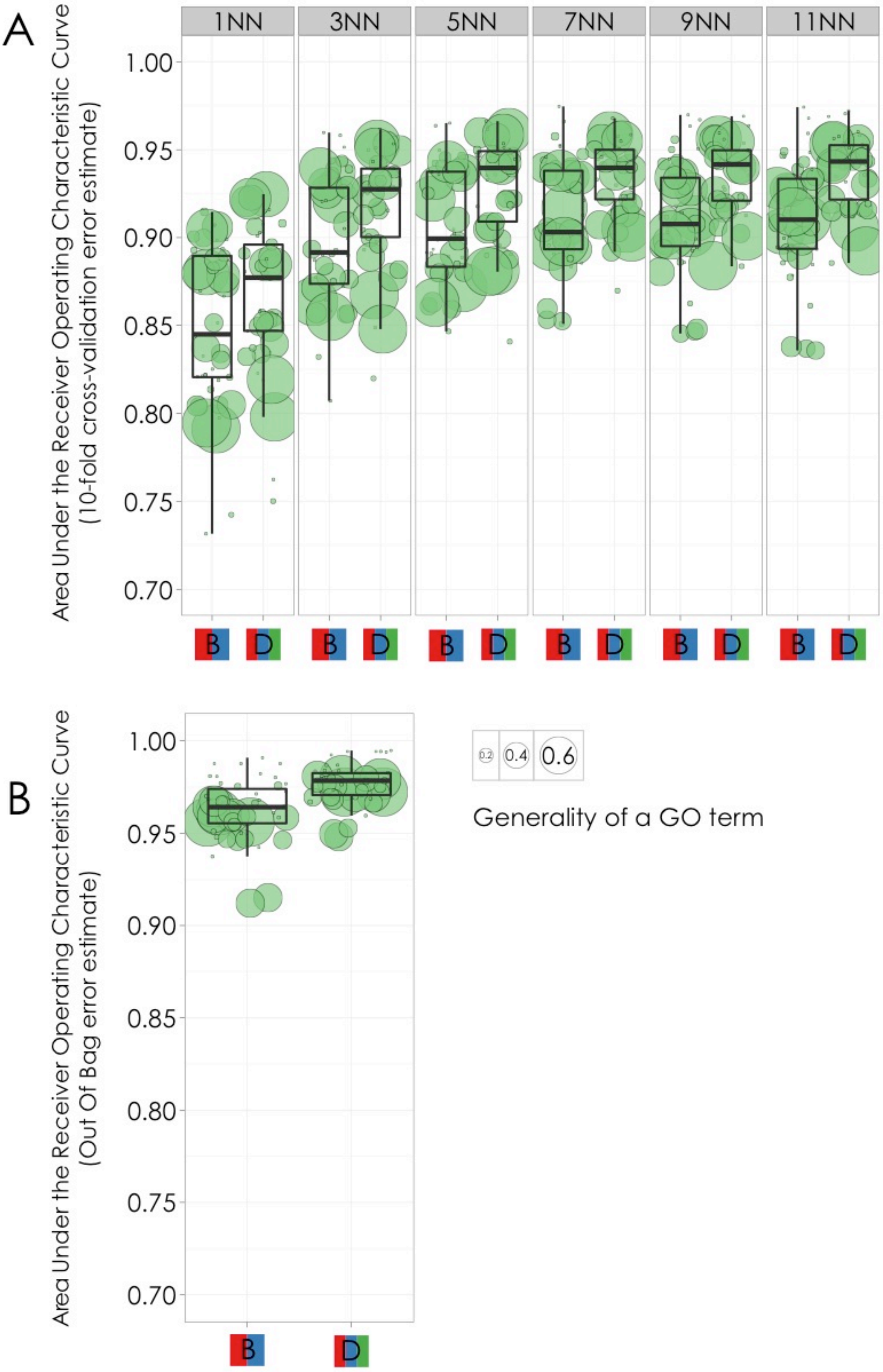
A
1NN
3NN
5NN
7NN
9NN
11NN
1.00
0.95
0.90
0.85
0.80
0.75
0.70
Area Under the Receiver Operating Characteristic Curve
(10-fold cross-validation error estimate)
B
D
B
Area Under the Receiver Operating Characteristic Curve
(Out Of Bag error estimate)
0.2
0.4
0.6
Generality of a GO term

Figure S9 (previous page): Predictive performance of the annotation models compared with the Area Under the Receiver Operating Characteristic Curve (AUC) for the Cellular Component ontology. A) Annotation model inferred using the kNN classifier: each panel shows the data for the k value denoted in the panel header. B) Annotation model built using the Clus-HMC classifier. The x axis represents the data used to infer the models: phylogenetic profiles are based on (C) OMA cliques of orthologs and OMA inferred orthologs and (D) OMA cliques of orthologs, OMA inferred orthologs, and OMA inferred paralogs. The y-axis represents the AUC. Each disc represents one GO term; its colour represents the ontology, while the area of the disc is proportional to the generality of the GO term: the frequency of the GO term among all annotations available in 07-02-2012 UniProt-GOA release. Each boxplot summarizes AUC for the dataset indicated on the x-axis. Lower, mid, and upper horizontal lines denote the first quartile, median and the third quartile, respectively; vertical lines reach 1.5 interquartile range from the respective quartile or the extreme value, whichever is closer.

References

Roth, A. C. J., Gonnet, G. H., & Dessimoz, C. (2008). Algorithm of OMA for large-scale orthology inference. *BMC bioinformatics*, *9*(1), 518. doi:10.1186/1471-2105-9-518

Škunca, N., Altenhoff, A., & Dessimoz, C. (2012). Quality of Computationally Inferred Gene Ontology Annotations. (L. J. Jensen, Ed.)*PLoS Computational Biology*, *8*(5), e1002533. Public Library of Science. doi:10.1371/journal.pcbi.1002533
